# Evaluation of a Circadian Rhythm and Sleep-Focused Mobile Health Intervention for the Prevention of Accelerated Summer Weight Gain Among Elementary School–Age Children: Protocol for a Randomized Controlled Feasibility Study

**DOI:** 10.2196/37002

**Published:** 2022-05-16

**Authors:** Jennette P Moreno, Hafza Dadabhoy, Salma Musaad, Tom Baranowski, Debbe Thompson, Candice A Alfano, Stephanie J Crowley

**Affiliations:** 1 Children’s Nutrition Research Center Department of Pediatrics-Nutrition Baylor College of Medicine Houston, TX United States; 2 Sleep and Anxiety Center of Houston, Department of Psychology, University of Houston Houston, TX United States; 3 Biological Rhythms Research Laboratory, Department of Psychiatry and Behavioral Sciences, Rush University Medical Center Chicago, IL United States

**Keywords:** summer, circadian rhythms, sleep, child obesity, elementary school

## Abstract

**Background:**

The i♥rhythm project is a mobile health adaptation of interpersonal and social rhythm therapy designed to promote healthy sleep and behavioral rhythms among 5-8-year olds during summer for the prevention of accelerated summer weight gain.

**Objective:**

This pilot study will examine the feasibility, acceptability, and preliminary efficacy of the i♥rhythm intervention. This will ensure that the research protocol and procedures work as desired and are acceptable to families in preparation for the fully powered randomized controlled trial. The proposed study will examine the willingness of participants to participate in the intervention and determine whether modifications to the intervention, procedures, and measures are needed before conducting a fully powered study. We will assess our ability to (1) recruit, consent, and retain participants; (2) deliver the intervention; (3) implement the study and assessment procedures; (4) assess the reliability of the proposed measures; and (5) assess the acceptability of the intervention and assessment protocol.

**Methods:**

This study will employ a single-blinded 2-group randomized control design (treatment and no-treatment control) with randomization occurring after baseline (Time 0) and 3 additional evaluation periods (postintervention [Time 1], and 9 months [Time 2] and 12 months after intervention [Time 3]). A sample of 40 parent-child dyads will be recruited.

**Results:**

This study was approved by the institutional review board of Baylor College of Medicine (H-47369). Recruitment began in March 2021. As of March 2022, data collection and recruitment are ongoing.

**Conclusions:**

This study will address the role of sleep and circadian rhythms in the prevention of accelerated summer weight gain and assess the intervention’s effects on the long-term prevention of child obesity.

**Trial Registration:**

ClinicalTrials.gov NCT04445740; https://clinicaltrials.gov/ct2/show/NCT04445740.

**International Registered Report Identifier (IRRID):**

DERR1-10.2196/37002

## Introduction

Accelerated summer weight gain has been shown to contribute to increasing rates of overweight and obesity during elementary school [1,2]. However, school-summer differences in diet, physical activity, and sedentary behavior have not been associated with differences in the rate of BMI change during the school year and summer [3]. While summertime interventions targeting children’s physical activity during summer have been demonstrated to increase physical activity levels, their impact on children’s BMI outcomes remains less clear [4]. These findings underscore the need to consider novel determinants of accelerated summer weight gain in the designs of interventions [5].

The transition from the school year to summer represents times during which students experience changes in their behavioral rhythms and routines [6]. Circadian rhythm misalignment has been implicated as a risk factor in obesity onset [7-9] and may be part of this seasonal/school-year variation. Specifically, shifts in the daily behavioral rhythms of sleep [10-13] are associated with increased adiposity, mediated through subsequent desynchronization between the central circadian clock in the suprachiasmatic nucleus and peripheral clocks [7,9,14]. School provides a natural structure for families, requiring children to follow a relatively consistent routine, promoting consistent sleep-wake patterns, mealtimes, and physical activity patterns. During out-of-school times such as summer vacation, bedtime is often later [15-17]. Later sleep timing may contribute to shortened sleep duration among younger elementary school-age children who fail to compensate with a delayed wake time [15]. Both shortened and more variable sleep duration have been associated with increased obesity risk in elementary-age children [10,12,18,19].

Differences in children’s sleep, physical activity, and light exposure during the school year and summer were related to children going to bed 1.5 hours later in summer compared with the school year [17]. Having a later sleep midpoint during summer predicted greater increases in BMI during summer, but was not associated with a change in BMI during the school year. Additionally, greater levels of outdoor light exposure during the school year predicted smaller increases in BMI during the school year. Interestingly, sleep duration, day-to-day variability in the midpoint between sleep onset and offset, physical activity levels, and sedentary behavior were not associated with a change in BMI during the school year or summer [17]. However, the importance of sleep timing and light exposure to changes in children’s BMI during the school year and summer suggests the potential importance of circadian rhythm–related behaviors, such as sleep timing and light exposure, for promoting a healthy weight status in children. To our knowledge, there is a lack of interventions designed to promote healthy sleep and behavioral rhythms among children during summer.

Interpersonal and social rhythm therapy (IPSRT) is an evidence-based treatment for bipolar disorder that promotes regularity of behavioral rhythms, such as sleep-wake cycles, meal times, and physical activity, to prevent desynchronization of endogenous circadian rhythms that precede depressive and manic episodes [20]. Behavioral rhythms are self-monitored, and behavioral strategies are used to increase the consistency of routines [20-22]. IPSRT is based on social zeitgeber theory [23], which posits that work schedules, family life, and community act as cues affecting circadian entrainment. These influences can be characterized as social demands, which influence circadian entrainment or the synchronization of circadian rhythms through “gating” exposure to the light-dark cycle and influencing the timing of behavioral rhythms [24]. It is hypothesized that a change in social demands such as the transition from the school year to the summer environment may lead to a change in behavioral rhythms [25,26], such as the timing of sleep, meals, and evening screen time, resulting in a change in exposure to morning and evening light as well as increased day-to-day variability. These changes in exposure may result in circadian misalignment, thus predisposing individuals to obesity [25-27]. Adapting IPSRT to promote healthy sleep and behavioral rhythms in children may offer promise as a method to prevent accelerated summer weight gain among children during summer.

The i♥rhythm project is an adaptation of IPSRT. It aims to promote healthy sleep habits and stable behavioral rhythms during summer for the prevention of obesity in young elementary school-age children. The intervention is designed to be delivered via mobile health (mHealth) technologies to better reach parents during summer. Parents receive information about the importance of healthy sleep and consistent behavioral rhythms and are guided through a series of steps to develop plans to support their child’s healthy sleep habits and stable behavioral rhythms during summer. This paper describes the research design, methods, and data analysis plan for an ongoing randomized controlled trial designed to evaluate the feasibility, acceptability, and preliminary efficacy of the i♥rhythm project to prevent accelerated summer weight gain. It is hypothesized that a priori feasibility criteria will be met and the intervention and assessment protocol will be found to be acceptable by participants (feasibility and acceptability criteria are outlined in detail in the “Methods” section). As this is a feasibility study, it is not powered to detect differences between groups; however, we anticipate that participants in the treatment condition will have an earlier circadian phase as measured by dim light melatonin onset (DLMO) and changes in BMI will be in the expected direction. Specifically, after intervention, participants in the i♥rhythm project will demonstrate earlier DLMOs and smaller increases in BMI compared with participants in the control condition.

## Methods

### Study Design

**Figure 1:**
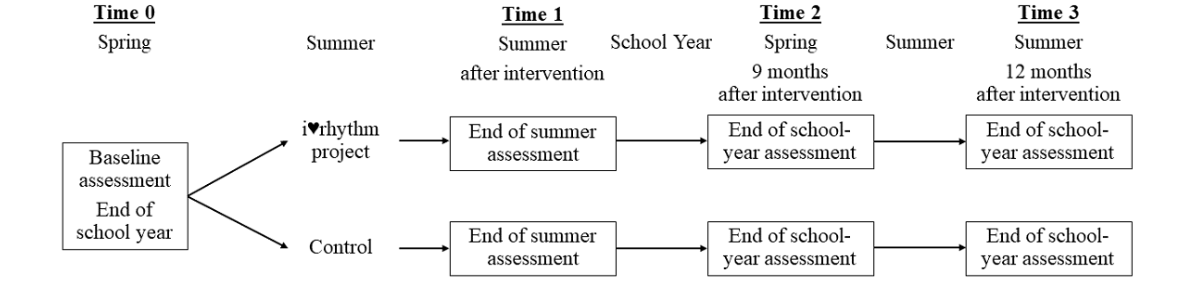
Study timeline.

Figure 1 illustrates the study timeline. We will employ a single-blind 2-group randomized control design (treatment and control) with randomization occurring after baseline in the spring/end of the school year (Time 0) and 3 postintervention evaluation periods: immediately after intervention (end of summer; Time 1), 9 months after intervention (spring; Time 2), and 12 months after intervention (summer; Time 3). Because the primary objective of the i♥rhythm project is to prevent children from beginning a trajectory toward overweight/obesity in elementary school, we will explore the impact of the intervention on change in BMI during the following summer (Times 2-3), identifying whether a maintenance intervention is needed. Following the intervention (Time 1) and final data assessment (Time 3), qualitative interviews will explore the acceptability of the intervention, barriers, facilitators, difficulties with study procedures, maintenance of improvements, and self-efficacy to maintain improvements.

### Participants

The i♥rhythm project is intended for children between the ages of 5 and 8 and their parents. This age group was selected because by using group-based trajectory modeling, we identified that about 19% of children began a trajectory toward overweight/obesity beginning the summer after kindergarten and the summer after second grade [28]. To be eligible for participation parent-child dyads must meet the following criteria: (1) index child must be between 5 and 8 years old and enrolled in kindergarten, first, or second grades; (2) index child must have a BMI >50th percentile (BMI>50th percentile in elementary school is associated with greater risk of transitioning to an unhealthy weight status or obesity at age 12 [29]); (3) parent identifies as a daily user of social media and willing to engage in a social media intervention on Facebook; (4) parent owns a smartphone; (5) parent is comfortable participating in the intervention and responding to questionnaires in English; (6) families must live within the Greater Houston area and be willing to attend 4 in-person assessment visits at the Children’s Nutrition Research Center (CNRC). Exclusion criteria include parent report that the index child (1) has been diagnosed with a chronic medical condition that influences sleep, eating behaviors, weight status, or circadian rhythms; (2) is being treated with a medication or supplement known to affect sleep, such as melatonin or stimulants; (3) has been retained 2 or more grades for academic reasons or has intellectual difficulties that would influence their ability to complete questionnaires or participate in interviews; (4) has participated in an obesity prevention or obesity treatment program in the last 6 months.

### Sample Size

Sample size recommendations for pilot studies vary, with some recommending 30 per group and others suggesting a few as 12 per group [30-33]. Our intention at the outset of the trial was to recruit 30 parent-child dyads per group. To account for an attrition rate of up to 20% [34,35] we aimed to recruit 36 parent-child dyads per group. The trial was intended to begin in the Spring of 2020, but due to the COVID-19 pandemic, the start was delayed by 1 year. Because of a delay in the trial start date, our recruitment goals were amended. Given the shortened study timeline, the new recruitment goal was to recruit a sample size of 16 parent-child dyads per group. Accounting for an attrition rate of up to 20%, this established a new recruitment target of 20 parent-child dyads per group (40 total parent-child dyads).

### Procedures

#### Recruitment

Recruitment began in March 2021. Because this study will examine the feasibility of a summertime intervention, parent-child dyads will be recruited only in the spring school semester, according to the Houston Independent School District Academic Calendar (approximately February through May). Participants will be recruited through a volunteer database and Facebook advertisements targeting families in the local Houston, Texas area. Data collection will not occur within 1 week following the transition to Daylight Saving Time [36].

#### Eligibility Assessment and Consent

Interested families will complete an online eligibility questionnaire that is accessed either through an email or a Facebook advertisement. To minimize in-person visits during the pandemic, families that completed the online questionnaire and were eligible will attend a virtual screening visit using Zoom to confirm their eligibility. Before the virtual visit, families will be provided a copy of the consent form and a video with instructions for collecting height and weight in the home [37]. If a digital scale or a measuring tape/ruler is not available in the home, one will be mailed to families. On the Zoom call, informed consent will be obtained, and parents will complete the at-home assessment of height and weight to verify that the child’s BMI is >50th percentile. Eligible families will then be scheduled for a baseline assessment at the laboratory that will take place between April and the commencement of the summer break from school. Time 1 assessment will begin following the conclusion of the 5-week intervention with the goal of completing all assessments during the remaining summer break before returning to school. Time 2 and 3 assessments will occur 9 months (end of the school year) and 12 months (end of summer) after completion of the intervention, respectively (Figure 1 and Table 1).

**Table 1:** Measurement timeline.

		Time 0	Intervention	Time 1	Time 2	Time 3
**Primary outcomes**						
	Recruitment goals	✓	✓			
	Intervention fidelity		✓			
	Intervention adherence		✓			
	Acceptability of assessments			✓		✓
	Treatment acceptability			✓		
	Retention		✓	✓	✓	✓
	BMI	✓		✓	✓	✓
	Dim light melatonin onset	✓		✓	✓	✓
**Secondary outcomes**						
	Sleep as assessed by actigraphy	✓		✓	✓	✓
	Body composition	✓		✓	✓	✓
	Dietary assessment	✓		✓	✓	✓
	Physical activity/inactivity	✓		✓	✓	✓
**Covariates**						
	Parent-child demographics	✓				
	General and sleep-related parenting practices	✓		✓	✓	✓
	Perceived stress (parent)	✓		✓	✓	✓
	Social support (parent)	✓		✓	✓	✓
	Child care during summer	✓		✓	✓	✓

#### Randomization

Following the baseline visit, parent-child dyads will be randomized to conditions (experimental or control). Because sleep duration has been shown to differ by sex [38,39], obesity status [10,40], and socioeconomic status (SES) [41], participants will be randomized to condition using a stratified permuted blocks procedure programmed by our biostatistician (SM) in SAS version 9.4 (SAS Institute Inc.). Participants will be identified as being male/female (according to sex at birth), healthy weight (ie, BMI percentile <85th percentile) versus nonhealthy weight (ie, BMI percentile ≥85th percentile), and middle/low SES versus high SES. SES will be estimated using a composite score using the family income-to-needs ratio and parent education level [41] (described in detail in the “Demographics” section).

#### Blinding

The principal investigator (PI; JPM) and statistician will be blinded to the treatment condition. The outcome assessors will not be blinded to the condition. Selection bias in the unblinded study personnel will be minimized through the randomization procedure.

### Conditions

#### Experimental Condition

The i♥rhythm project comprises five 15-minute sessions focused on (1) having consistent daily bedtimes; (2) providing opportunities for sunlight exposure during the day and minimizing exposure to artificial light at night; (3) providing opportunities for activity during the day so that the child is ready to fall asleep at night; (4) ensuring the last bite of food is 1-2 hours before bed; and (5) reviewing and developing of a maintenance plan.

Self-determination theory was used to guide adaptation of the behavior change components of the intervention [42]. Self-determination theory aims to promote sustained behavior change by emphasizing intrinsic motivation, in this case promoting the satisfaction of 3 basic psychological needs: autonomy (independence to choose to change behavior), competence (belief in one’s ability to perform a behavior), and relatedness (alignment with one’s core beliefs and values) [42].

**Figure 2:**
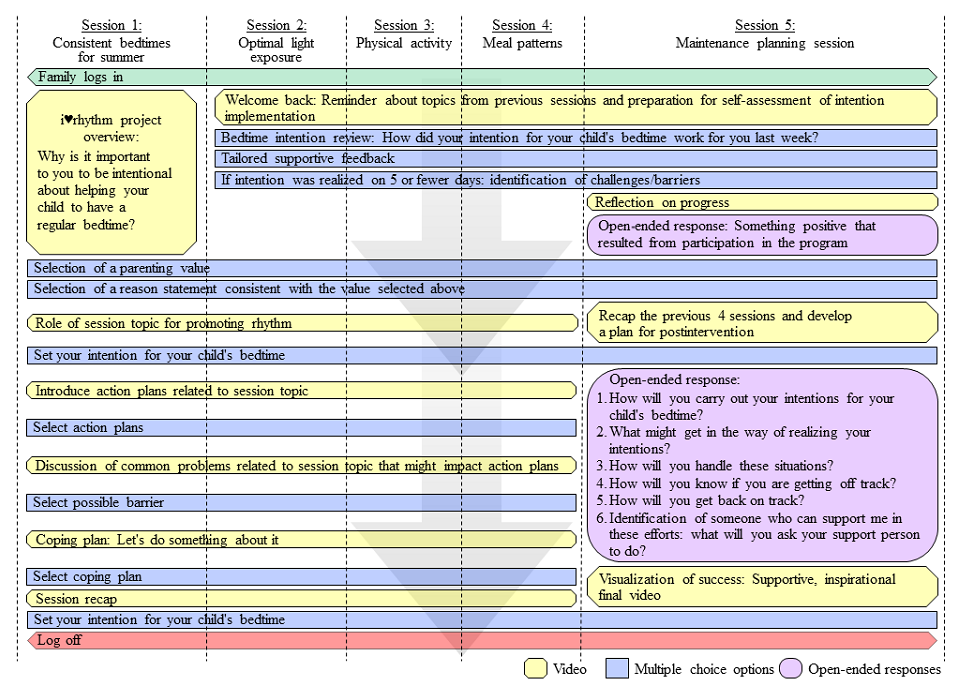
Intervention framework.

The 5-week session intervention framework and flow are presented in Figure 2. The first 4 sessions will each involve a series of videos and multiple-choice questions that guide parents through the following: (1) identification of a value that is important to them as a parent, such as having a healthy child, being spiritual, being a role model, being responsible; (2) identification of a reason why they might want to encourage their child to have a regular bedtime that is consistent with the value they selected; (3) education regarding the topic of the week and relevance to promoting a consistent bedtime; (4) implementation intention in which parents set their intention for their child’s bedtime that week and make an action plan for how they would achieve that bedtime goal utilizing skills that were introduced in the educational video; (5) identification of potential barriers to achieving their action plan; and (6) development of a coping plan in the event they encounter the identified barriers. The final screen will contain a summary of their responses, links to additional resources, and an option to print the plan (Multimedia Appendix 1). In between sessions parents will monitor and record their child’s bedtime. Daily light exposure, physical activity, and whether the child’s last bite of food was 1-2 hours before bedtime will be monitored on an intermittent basis beginning the week the topic is introduced. The fifth session will review the 4 topics and guide families through the development of a maintenance plan using open-ended questions. A summary of the developed maintenance plan is provided to participants at the end of the session. The sessions have been developed and programmed on an online survey platform called Alchemer [43].

Considering the developmental abilities of children aged 5-8, parents are considered the primary agent of change, though child viewing of the videos is encouraged. Session links will be emailed to participants and posted to a private Facebook group on Sunday mornings beginning the first week of summer break. The private Facebook group serves to promote social support for parents and to provide parents access to additional resources through daily (Monday-Friday) posts (Table 2 contains an example week). The Facebook group will be administered and monitored daily by research staff who assisted in the development of the intervention. The PI (JPM) will meet weekly with the intervention delivery team to ensure the intervention is delivered according to protocol. To ensure the PI remains blinded, no participant identifying information will be used in these meetings.

**Table 2:** Example week of Facebook posts.

Day	Themes	Topic	Text of the post
Sunday	Intervention Session	Session 1—Consistent Bedtimes	*Today's the day! Click on the link below to begin Session 1 of the i♥rhythm project. #sleep #consistencyiskey #bedtimeroutine*
Monday	Topic Introduction	Setting Up a Calming Bedtime Routine	*With long days and busy family schedules, how do you set up a calming bedtime routine for your child? Read this resource to learn more (link). If you have any issues accessing the Dropbox folder, please let us know. #sleep #bedtime routine*
Tuesday	General	Reminder About Resources	*Just a quick note that additional resources can be found in our Box folder (link). Over the next few weeks, we will be covering the following topics: Consistent Bedtimes, Light Exposure, Physical Activity, Meal Timing, and Maintenance. Remember to reach out if you have any questions! #resources*
Wednesday	Barriers and Solutions	Helping Your Child Fall Asleep Independently	*Is your child having trouble falling asleep independently? Click on the link to read about strategies to get your child's sleeping schedule back on track. If you have additional strategies to share, feel free to comment with them below!*
Thursday	Status Update	Check-in poll about progress	*What are some strategies that helped your child keep a consistent bedtime this week? For the poll, you can choose multiple options, add your own, or type it in the comments. Note: the results of the poll are not anonymous.*

#### Control

Control participants will not receive an intervention and will only participate in Time 0, 1, 3, and 4 assessments as outlined in Table 1.

### Process Evaluation

Process evaluation documents intervention delivery and provides insight into the mechanisms by which programs work [44]. Following the framework of Baranowski and Jago [45], we will assess recruitment of participants, maintenance of participation, the context of implementation, resources necessary for implementation, implementation (completion of sessions, daily diaries, and engagement in social media), reach, barriers to implementation, and exposure to the program. Participant completion of sessions will be monitored via logins, video views, and completion of interactive content and diary data. Engagement in social media will be collected via manual abstraction from the newsfeed of the private group [46]. Other issues such as acceptability will be assessed by questionnaires after intervention. Postassessment interviews will examine (1) acceptability of the interventions, (2) perceived benefits of participation, (3) barriers to participation, (4) reasons for drop out. Following the intervention (Time 1) and final data assessment (Time 3), qualitative interviews will be conducted. Interviews will use a standardized script, open-ended questions, follow-up questions, prompts, and areas to probe. Interviews will be conducted until theoretical saturation is achieved [47,48]. Thematic data analysis will identify common themes and patterns [49]. Each statement will be assigned a broad category and assessed for meaning units, specific categories, and subcategories. These will be compared and contrasted by gender, overweight status, and SES level.

### Adherence

Participant completion of sessions will be monitored by an outcome assessor (HD) who will send SMS text message reminders to the family if they did not complete a session within 2 days after the release of data. Families who miss a session will be sent the next session the following week. Facebook post views and engagement will also be monitored using metrics provided by Facebook.

### Data Collection

Two weeks before the scheduled assessments, actigraphs will be mailed to the child’s home. A link to an online instructional video will demonstrate proper wear and how to avoid covering the accelerometer with clothing [50]. Children will wear actigraphs (GT3X-BT; ActiGraph, LLC) on the wrist of their nondominant hand to assess their sleep and activity for 7 days and 8 nights during the school year. Parents will complete a daily sleep diary and monitor-wear logs to record the times the accelerometer is removed and the reason for removal [51-53]. A paper copy of these forms will also be provided for note-taking purposes. Parents will download an app called Centrepoint (ActiGraph, LLC) to their phone and will be asked to use this app to upload their child’s actigraph data to the cloud via a Bluetooth connection. Download instructions will be emailed to participants and research staff will follow-up to provide a PIN. During this follow-up, the parent will perform a preliminary upload to verify data are transferring. The Centrepoint app will allow research staff to monitor participants’ compliance with accelerometer wear and ensure sufficient wear time is achieved before coming into the sessions. Sufficient wear time will be defined as having at least five nights [51] and days of activity data. Valid days will be defined as at least ten hours of wear time in 24 hours [54]. As sleep will not be scored until after the actigraphs are returned, this determination will be made by research staff using visual inspection of the Centrepoint data. During the 8 days when the monitor is worn, parents will be asked to complete a web-based dietary food recall using the Automated Self-Administered 24-Hour Dietary Assessment Tool (ASA24) 3 times and complete a series of questionnaires (described below). Completion of the ASA24 will be monitored by the research staff. If the online diary status shows as incomplete, the parent is emailed the next day to complete another day of intake until 3 full days are complete. The aim is to obtain dairy intake data for 2 weekdays and 1 weekend day.

Approximately 1-2 days following the eighth day of accelerometer wear, assessment visits will be scheduled. Participants will be scheduled to arrive at the laboratory at least six hours before their typical bedtime. Typical bedtime will be estimated using visual inspection of the actigraphy data in Centrepoint along with parent sleep diaries. On the day of the laboratory visit, participants will be asked to avoid intake of caffeine, chocolate, nonsteroidal anti-inflammatory drugs, and cannabidiol products. Upon arrival at the laboratory, anthropometric assessments will be conducted and actigraph data will be downloaded. Children and their parents will be taken to a dimly lit (<5 lux) circadian phase assessment suite (a private room with a table and chairs and a futon chair with an adjoining bathroom). Following established procedures with children, saliva (~1 mL) samples will be collected using untreated Salivettes (Starstedt, Inc.) every hour beginning 5 hours before and ending 1 hour following typical bedtime. Before the collection of samples, children will be seated for 10 minutes to minimize postural effects on melatonin concentration. If participants eat or drink before the sample collection, they will gently brush their teeth with a soft-bristled toothbrush and water. Saliva samples will be centrifuged and frozen for later analysis of melatonin. Assessment visits will end after the final saliva sample collection.

To compensate participants for travel and the inconveniences associated with assessments requiring a minimum stay of 7 hours in the laboratory and keeping children awake past their bedtime at each assessment, participants will be offered US $100 at Times 0 and 1 and US $150 at Times 2 and 3 to encourage participation in assessments.

### Primary Outcomes

#### Feasibility Criteria

The feasibility of the study will be established by our ability to recruit the needed sample size and retain at least 80% of participants at Time 1 and 60% at Time 3. The feasibility of the intervention will be determined based on delivery of all components of the intervention as designed, favorable acceptability ratings by 80% of parents randomized to the experimental condition, completion of greater than 60% of daily self-monitoring, and views of 80% of intervention sessions by the experimental sample.

#### Treatment Acceptability

Participants assigned to the experimental condition will complete the Treatment Acceptability Report Form-Revised (TARF-R) at Time 1. The TARF-R is a 20-item global measure of treatment acceptability for behavioral interventions. Examples of items include (1) “How clear is your understanding of this intervention?”; (2) “How acceptable do you find the intervention to be for you and your child?”; (3) “How reasonable do you find the intervention to be?”; (4) “How likely is the intervention to make improvements in your child’s health habits?”. The TARF-R has demonstrated good internal consistency (αs>.69) and evidence of construct validity (reference). A favorable rating is considered 4 or greater.

#### Anthropometrics

While feasibility criteria will serve as a primary outcome for this study, change in BMI will serve as the primary outcome for the fully powered randomized controlled trial. BMI is the most common indicator of body size and has been consistently correlated with metabolic problems in children. Participants’ height and weight will be measured. Weights will be assessed in light clothing without footwear using a Healthometer digital scale. BMI (kg/m^2^) will be computed and BMI percentile and standardized BMI will be calculated from age and gender normative data. BMI percentile will determine the weight status group. Based on baseline BMI percentiles, children will be classified into 3 weight categories: healthy weight (5th percentile to <85th percentile for BMI), overweight (≥85th to <95th percentile for BMI), and obese (≥95th percentile for BMI). Change in BMI and standardized BMI are the best proxy measures for change in fat mass and standardized fat mass, respectively, and will assess exploratory hypotheses.

#### Circadian Phase

While feasibility criteria will serve as the primary outcome for this study, circadian phase will serve as a primary outcome for the fully powered randomized controlled trial. Circadian phase can be examined by measuring the timing of melatonin onset under dim light conditions (DLMO). Compared with markers of endogenous circadian rhythms, melatonin is relatively robust [55,56]. Salivary DLMO measures have demonstrated high intraclass correlations (0.93) with plasma and sensitivity and specificity comparable to plasma assays [57]. Saliva samples will be analyzed using radioimmunoassay test kits (NovoLytiX GmbH) at SolidPhase, Inc. The DLMO phase will be determined using linear interpolation across the melatonin concentration values detected in the saliva samples before and after concentration levels increase to and remain above 4 pg/mL [58,59]. The DLMO phase or the time at which melatonin levels rise and remain above 4 pg/mL will be used to compare differences in the circadian phase across conditions.

### Secondary Outcomes

#### Body Composition

Total body fat and percent body fat will be assessed using air-displacement plethysmography (BOD POD; COSMED USA, Inc.). Participants will enter the chamber wearing a 1-piece swimsuit and swimming cap. The procedure will be conducted twice for 45 seconds. If a difference greater than 150 mL between body volumes is detected, then a third measurement will be collected. The thoracic gas volume will be estimated using the BOD POD software (BOD POD GS-X) [60]. Body density will be calculated by dividing the raw body mass (kilogram) by the corrected body volume (liter). Fat-free mass, fat mass, and percent body fat will be derived from body density using the Siri equation [61].

#### Actigraphy

Actigraphs (GT3X-BT) worn on the wrist of the dominant hand 24 hours a day for 7 days will measure sleep duration, the timing of sleep onset, and waking as well as physical activity and light exposure. Using the Sadeh algorithm [62] epochs will be scored as sleep or wake. According to established protocols [17,63], each sleep episode reported in the parent diary will be inspected in the activity data. Nights will be considered valid if the participant provided 20 minutes of wear time before sleep onset. Nonwear time in the hour before bedtime must be less than 60 minutes unless confirmed by the wear log, or unless ambient light data are available to confirm bedtime. Sleep onset will be defined as the beginning of the first 3 consecutive epochs scored as sleep. Sleep offset will be defined as the last 5 consecutive minutes of sleep occurring before 15 minutes after the reported wake-up. Sleep midpoint will be defined as the midpoint between sleep onset and offset. Children’s physical activity will be measured using vector magnitude activity counts captured in 60-second epochs and categorized into sedentary, light, moderate, and vigorous physical activity using established cut points [64].

#### Dietary Assessment

The ASA24 [65] will be used to assess children’s dietary intake, including total daily caloric intake, the timing of intake, timing of the last eating episode of the day, and caloric intake of the last eating episode. As recommended, parents will complete the ASA24, providing a proxy report of their child’s dietary intake [66]. Investigators will use 3 days of diet assessment as this optimizes the prediction of doubly labeled water-estimated energy expenditure [67]. The ASA24 will be used to assess average daily caloric intake. Average caloric intake in the morning (6:00 AM to <10:00 AM) and nighttime (7:00 PM to <6:00 AM) will be calculated using defined criteria [68] along with the timing of the first and last eating episode of the day.

### Covariates

#### Demographic Information

Parents will report on their own and child’s date of birth, sex, ethnicity, and race. The SES will be assessed using the family income-to-needs ratio [69]. This metric considers the family income level in relation to the number of individuals supported by the income level [41]. Parents will report annual familial income according to the following categories: US $10,000 to US $20,000; US $20,000 to US $35,000; US $35,000 to US $50,000; US $50,000 to US $75,000; or more than US $75,000. The mean of the reported income level will be divided by the federal poverty threshold for a household of that size [69]. Ratios with a value less than 1 will be assigned a value of 1=1 (poverty), 1-2=2 (living near the poverty line), 2-3=3 (lower middle class), 4=4 (middle class or higher) [41]. Parent education will be reported according to the following categories: seventh grade or less, completion of eighth grade, ninth to eleventh grade, high-school graduate, partial college or specialized training, bachelor’s degree, or graduate degree. These education categories will be assigned scores on a scale of 1-7, respectively. A composite score will be created by adding together the income-to-needs ratio level with the parent education level to create a composite score [41]. Values of 8 or lower will be considered middle/low SES and values of 9 and 10 will be considered higher SES.

#### Parenting Structure

The Comprehensive General Parenting Questionnaire (CGPQ) is a parent report of parenting practices among parents of 5-13-year olds [70]. Investigators will assess subscales related to parenting structure (Inconsistent Discipline, Consistency, Organization, and Scaffolding). There is support for the construct validity of the CGPQ [70]. Parenting structure subscales have demonstrated acceptable internal reliability (ranging from 0.67 to 0.74) [70]. Subscale scores range from 5 to 25. Higher scores indicate higher levels of structure.

#### Bedtime Routines

The Bedtimes Routines Questionnaire (BRQ) is a 31-item parent report measure of children’s bedtime routines comprising 3 scales measuring the consistency of bedtime routines (weekday and weekend), reactivity to changes in bedtime routines, and frequency of adaptive and maladaptive activities [71]. The BRQ scales have acceptable internal consistency (α) ranging from .69 to .90. Scores on the Consistency and Adaptive Behavior subscales range from 10 to 50, with higher scores reflecting more consistent bedtime routines and higher. The Reactivity scale scores range from 5 to 25, with higher scores reflecting greater reactivity. The Maladaptive Behavior scale has possible scores ranging from 6 to 30 with higher scores reflecting more maladaptive behaviors [71].

#### Summer Care Arrangements

Children’s involvement in summer school, childcare, entertainment programs, as well as day or overnight camps will be assessed by a parent report survey based on a modified version of the Early Childhood Longitudinal Program Kindergarten Class’s parent interview on summer activities [72].

#### Stress (Parent)

The 10-item Perceived Stress Scale (PSS-10) is a self-report measure of the parent’s perceived stress with established acceptable psychometric properties (αs >.70, test-retest criterion coefficient >0.7, validated factor structure, and evidence of convergent validity) [73]. Scores range from 0 to 40, with higher scores reflecting higher levels of perceived stress.

#### Social Support (Parent)

Interpersonal Support Evaluation List (ISEL) is a 12-item self-report measure of the parent’s perceived availability of social support [74]. The “Tangible” subscale assesses perceived availability of resources and material aid; the “Appraisal” subscale assesses the perceived availability of another individual(s) to discuss one’s problems; the “Self-esteem” subscale assesses the perceived availability of someone to compare one’s self to in a positive manner; and the “Belonging” subscale assesses the perceived availability of having others to socialize and do things with. Scores range from 0 to 30 on each scale, with greater scores indicating higher levels of social support [74].

#### Treatment Motivation

Participants assigned to the experimental condition will complete13 items regarding their motivation to follow the procedures of the program and to remain in the program. Items were adapted from [75,76].

### Data Management

A manual of procedures, including protocols related to the collection of each measure and storage, was developed at the beginning of the study. In 2021, before the beginning of data collection, an in vivo training session was scheduled and all data collectors took turns practicing all assessment protocols, and feedback was provided by the PI (JPM) and senior coordinator (HD). The manual of procedures will be maintained and continually updated with input from all related study coordinators. Weekly meetings will be held with the PI and study coordinators to review recruitment, study progress, and data storage and management.

### Data Analysis Plan

A statistical analysis plan will be written before the database is locked and breaking the blind. The feasibility of the intervention will be assessed from staff-collected and parent-reported variables according to the previously stated criteria. We will examine the internal consistency of all questionnaires using Cronbach alpha. Decisions regarding the need for changes before further testing of the intervention will be made based on the attainment of feasibility criteria and postassessment interviews. This pilot study is not powered to detect differences in outcome variables and therefore drawing conclusions about the efficacy of the intervention based on statistically insignificant differences in change in BMI, standardized BMI, or circadian phase is inappropriate [30,77-79]. Descriptive statistics and confidence intervals will be used to examine mean differences and the effect sizes of change in outcome variables between conditions. Exploratory analyses will be used to estimate intervention effect over time using a repeated-measures regression approach while controlling for sex and SES, and accounting for the within-subject correlation and the nesting of children within families. The model will include a fixed effect for the repeated measurements (Times 0, 1, 2, and 3), a random subject effect, and treatment group as a between-subjects factor (i♥rhythm and control). Children’s differences across the groups in change in BMI, standardized BMI, and circadian phase will be tested using a group × time interaction. Change in BMI was included as an outcome because it is considered the preferred measure of change in adiposity among children over periods of less than 1 year [80-82]. Separate models will examine other dependent variables of interest including sleep duration, the timing of sleep onset, and interdaily similarity. Because the study is not powered to detect group differences in outcomes, effect sizes will be examined based on the standardized mean difference (Cohen *d*) criteria for small (0.20), medium (0.50), and large (0.80) [83,84].

### Ethics Approval

This study was approved by the institutional review board at Baylor College of Medicine (approval number H-47369).

## Results

This protocol was written following the SPIRIT (Standard Protocol Items: Recommendations for Interventional Trials) 2013 statement for clinical trial protocols [85,86]. The trial was supposed to begin in the Spring of 2020; however, the trial onset was delayed 1 year as new human subjects research was halted by Baylor College of Medicine in the spring of 2020 due to the COVID-19 pandemic. As a result, recruitment began in March 2021. As of March 2022, data collection and recruitment are ongoing. Data collection is anticipated to end in September 2023. Amendments to the protocol will be made on ClinicalTrials.gov. Results will be communicated via publication and ClinicalTrials.gov.

## Discussion

### Overview

Summer is a time during which children’s risk of obesity increases substantially [28,87]. Disrupted sleep patterns and behavioral rhythms, which may occur in the transition from the school year to summer vacation [6,15,16], have been implicated in the onset of obesity [7-9]. IPSRT is an evidence-based treatment for bipolar disorder that has demonstrated efficacy to prevent the reoccurrences of mood disturbances by preventing the misalignment of endogenous circadian rhythms through the promotion of stable behavioral rhythms (eg, sleep and meal patterns) [20]. The i♥rhythm project is an adaptation of IPSRT as an mHealth intervention to promote stable behavioral rhythms in children during summer vacation for the prevention of summer increases in BMI.

This study is a randomized controlled trial designed to evaluate the feasibility, acceptability, and preliminary efficacy of the i♥rhythm project to prevent accelerated summer weight gain. It is hypothesized that a priori feasibility criteria will be met and the intervention and assessment protocol will be found to be acceptable by participants. As this is a feasibility study, it is not powered to detect differences between groups; however, we anticipate that participants in the treatment condition will have circadian phases that trend toward being earlier and changes in BMI will tend to be smaller compared with participants in the control condition.

Strengths of this study include a rigorous design involving randomization, a comparator condition, and multiple follow-up assessments. Our hypotheses and outcomes have been stated a priori and criteria for evaluating the feasibility and acceptability of the study have been established. Additionally, procedures for process evaluation, intervention fidelity, and assessment of intervention adherence have been developed.

### Limitations

This is a feasibility study and is not powered to detect differences in circadian phase, sleep, or BMI outcomes. If deemed feasible in this study, a fully powered randomized controlled trial will be needed to determine the efficacy of this approach. The current research is being conducted during the COVID-19 pandemic. Due to the halting of new human subjects research in the spring of 2020, the trial onset was delayed by 1 year. The initial phase of recruitment (March-May 2021) began at the end of the second COVID-19 wave (2021) and Time 1 (postintervention assessments) ended just as the Delta wave was beginning in August 2021. For many children, the 2020-2021 school year may not have been typical. Many children may not have attended school in person, potentially weakening the effect of school-summer differences. The 2021-2022 school year has been more typical in the sense that most children returned to school in person. While there is evidence that the pandemic has exacerbated the obesity epidemic in children [88,89], the extent to which patterns of improvement in school-year weight outcomes and accelerated summer weight gain have persisted during the pandemic is unclear. Vaccines became available to 5-8-year olds in November of 2021. The next phase of recruitment will begin as the Omicron-fueled wave appears to be declining (February 2022). It is unclear the extent to which the pandemic has affected our ability to recruit and retain families in the study.

### Conclusions

This study seeks to prevent child obesity by combining 2 distinct intervention concepts (enhanced sleep and stable behavioral rhythms) that have previously not been combined for the prevention of childhood obesity. This represents a departure from traditional obesity prevention approaches that have focused on the simple energy balance of diet and physical activity [5]. The proposed research aims to expand our understanding of the role of sleep and behavioral rhythms in the prevention of childhood obesity and adapting IPSRT for children should result in an innovative evidence-based approach to promoting stable behavioral rhythms for obesity prevention. In addition, this study focuses on summer, a time when young children experience significant increases in BMI [90,91]. To our knowledge, the proposed research is the first to (1) provide preliminary evidence regarding the impact of an intervention for the prevention of increases in BMI during summer and the longer-term impact on weight status, (2) combine 2 distinct theories regarding the obesogenic role of sleep and behavioral rhythms among children, and (3) examine a novel adaptation of an evidence-based treatment of bipolar disorder for the prevention of childhood obesity.

## Appendix

Multimedia Appendix 1 Intervention screenshots.

Multimedia Appendix 2 Peer-reviewer report from the Pediatrics Subcommittee - Eunice Kennedy Shriver National Institute of Child Health and Human Development (CHHD-A) Initial Review Group (National Institutes of Health, USA).

## Abbreviations

ASA24: The Automated Self-Administered 24-Hour Dietary Assessment Tool
BRQ: Bedtimes Routines Questionnaire
CGPQ: Comprehensive General Parenting Questionnaire
CNRC: Children’s Nutrition Research Center
DLMO: dim light melatonin onset
IPSRT: interpersonal and social rhythm therapy
ISEL: Interpersonal Support Evaluation List
mHealth: mobile health
PI: principal investigator
PSS-10: 10-item Perceived Stress Scale
SES: socioeconomic status
SPIRIT: Standard Protocol Items: Recommendations for Interventional Trials
TARF-R: Treatment Acceptability Report Form-Revised
