# Supplementary figures and images for: Evaluation of a Circadian Rhythm and Sleep-Focused Mobile Health Intervention for the Prevention of Accelerated Summer Weight Gain Among Elementary School–Age Children: Protocol for a Randomized Controlled Feasibility Study
Source: JMIR Res Protoc. 2022 May 16;11(5):e37002. doi: 10.2196/37002 (PMC9152728; doi:10.2196/37002)

## Slide 1
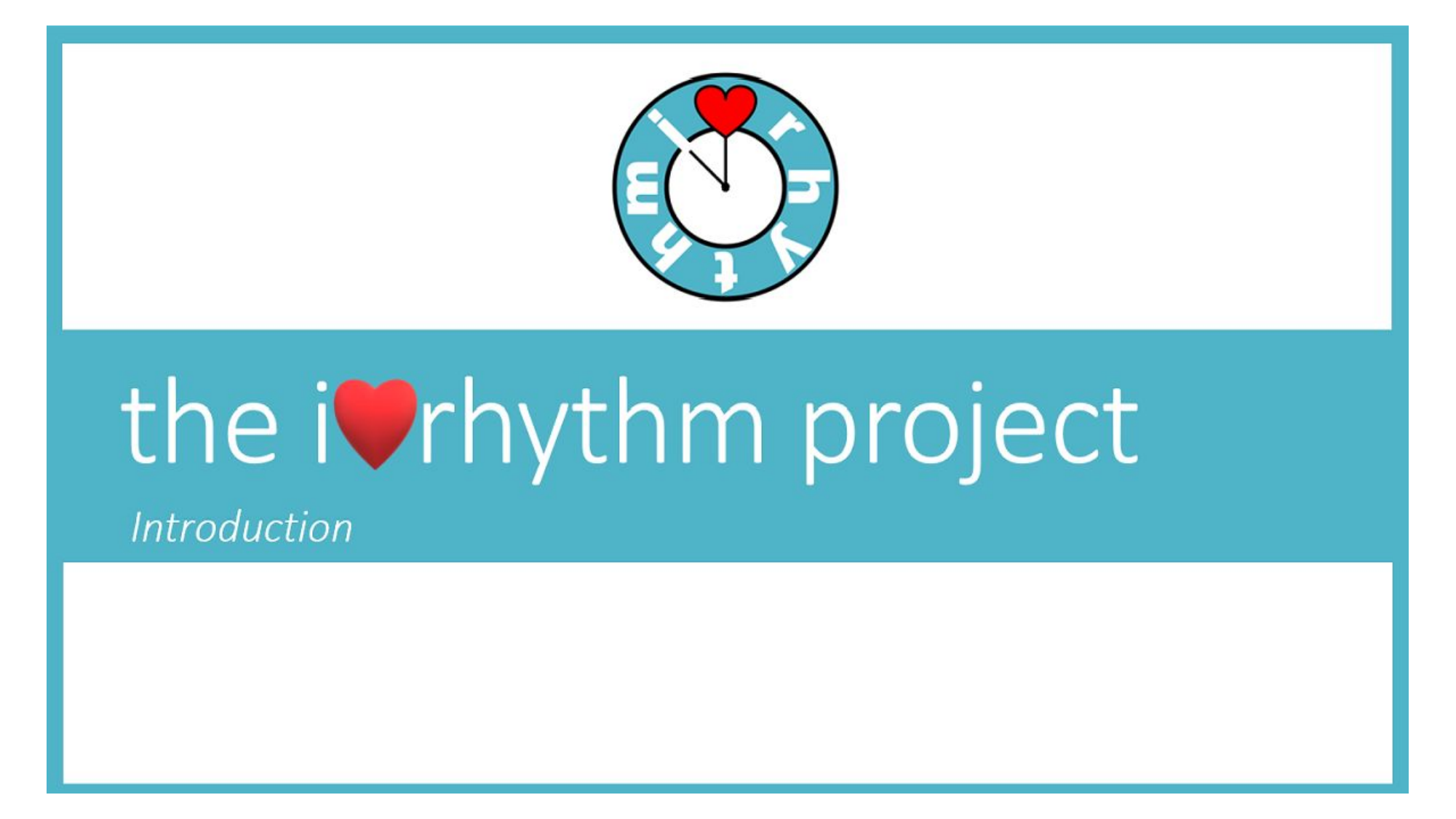

## Slide 2
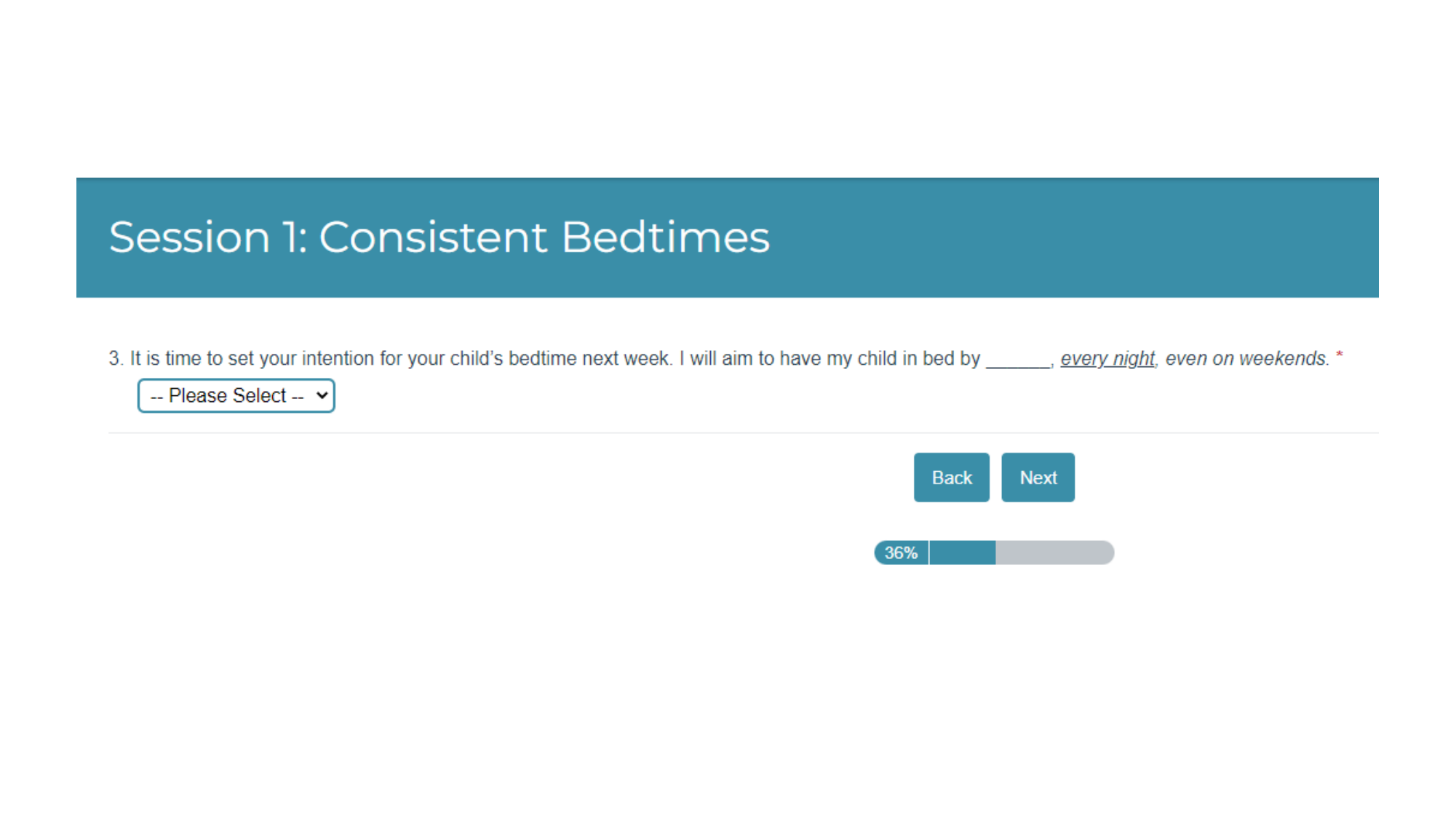

## Slide 3
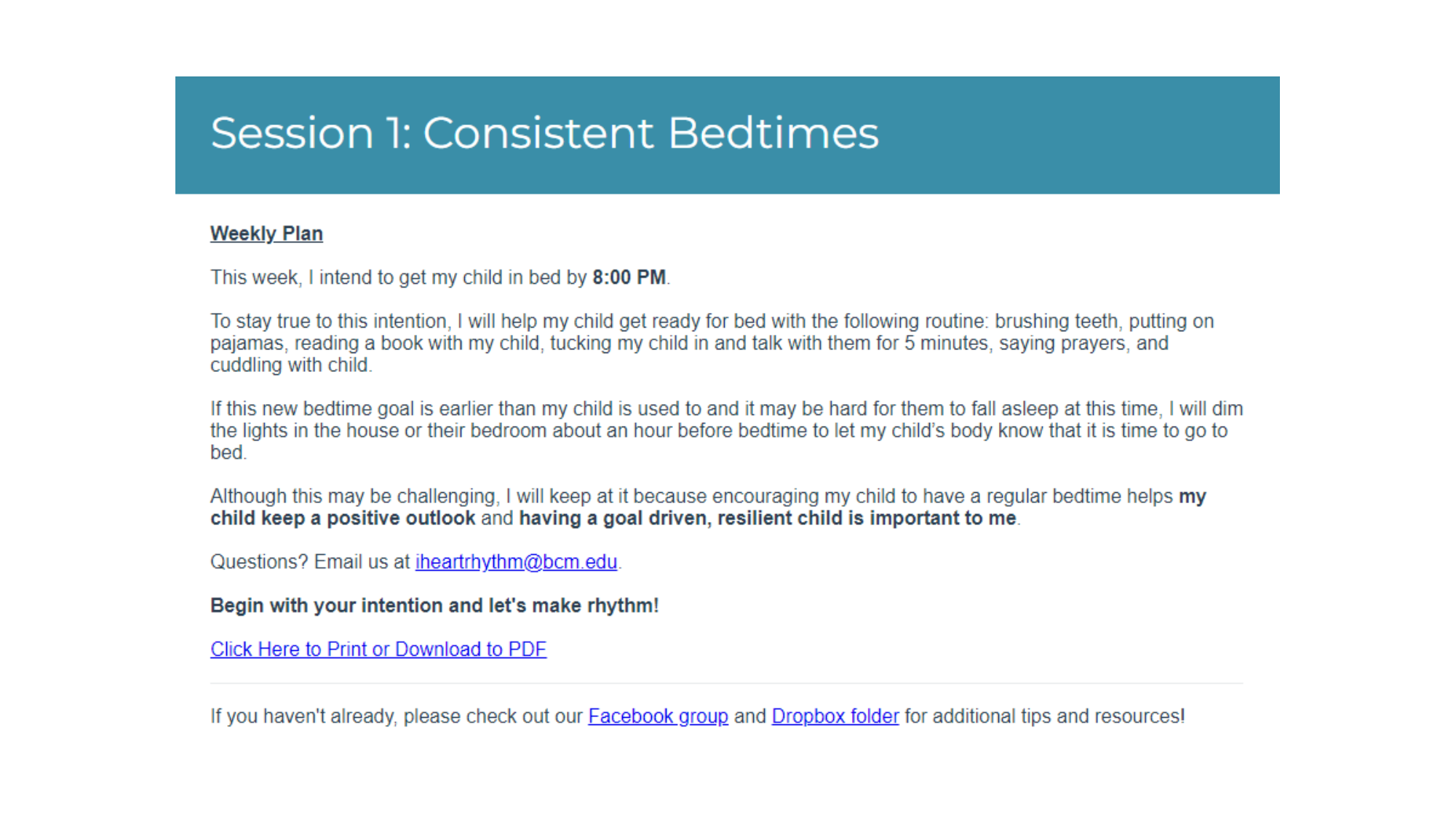

Supplement: Multimedia Appendix 1 [file resprot_v11i5e37002_app1.pptx]
