# Supplementary material for: Evaluation of a Circadian Rhythm and Sleep-Focused Mobile Health Intervention for the Prevention of Accelerated Summer Weight Gain Among Elementary School–Age Children: Protocol for a Randomized Controlled Feasibility Study
Source: JMIR Res Protoc. 2022 May 16;11(5):e37002. doi: 10.2196/37002 (PMC9152728; doi:10.2196/37002)
Supplement: Multimedia Appendix 2 [file resprot_v11i5e37002_app2.pdf]

SUMMARY STATEMENT
(Privileged Communication)

PROGRAM CONTACT:
LAYLA ESPOSITO
(301) 435-6888
espositl@mail.nih.gov

Release Date: **12/15/2016**
Revised Date:

Application Number: **1 K99 HD091396-01**

Principal Investigator

MORENO, JENNETTE P

Applicant Organization: BAYLOR COLLEGE OF MEDICINE

Review Group: **CHHD-A**
Pediatrics Subcommittee

Meeting Date: **10/20/2016**
Council: **JAN 2017**
Requested Start: **04/01/2017**

RFA/PA: **PA16-193**
PCC: **CDBB -LE**

Project Title: **The i♥Rhythm Project: Healthy sleep and behavioral rhythms for obesity prevention**

SRG Action: **Impact Score:24**
Next Steps: **Visit http://grants.nih.gov/grants/next_steps.htm**
Human Subjects: **30-Human subjects involved - Certified, no SRG concerns**
Animal Subjects: **10-No live vertebrate animals involved for competing appl.**
Gender: **1A-Both genders, scientifically acceptable**
Minority: **1A-Minorities and non-minorities, scientifically acceptable**
Children: **1A-Both Children and Adults, scientifically acceptable**
Clinical Research - not NIH-defined Phase III Trial

Project Year	Direct Costs Requested	Estimated Total Cost
1	102,602	104,356
2	104,617	106,406
3	249,000	253,258
4	249,000	253,258
5	249,000	253,258
TOTAL	954,219	970,536

ADMINISTRATIVE BUDGET NOTE: The budget shown is the requested budget and has not been adjusted to reflect any recommendations made by reviewers. If an award is planned, the costs will be calculated by Institute grants management staff based on the recommendations outlined below in the COMMITTEE BUDGET RECOMMENDATIONS section.

RESUME AND SUMMARY OF DISCUSSION: This is a K99 application from Dr. Jannette Moreno proposing to study the healthy sleep and behavioral rhythms for obesity prevention. This is a very well formulated and written proposal from an enthusiastic and productive investigator working at Baylor College of Medicine. With training in clinical psychology, she has served for many years as research and project coordinator, now she needs more training to make her transition to independent investigator possible. The research objective of this proposal is to evaluate interventions to prevent summer increases in BMI in children. The research plan has several weaknesses and some major weaknesses like failure to account for confounding factors. The team of mentors is impressive, and she already published with some of them. The career plan is adequate for this stage of her career. She is expected to be successful in her career path and contribute valuable research to the field of obesity prevention in children. However, due to the weaknesses in research plan the strengths of this application did not clearly outweigh its weaknesses.

DESCRIPTION (provided by applicant): Increases in children's standardized BMI (BMIz) have been demonstrated during summer across the US and internationally. The largest study to demonstrate this was our 5-year longitudinal study (kindergarten – 5th grade) which demonstrated that beginning in early elementary school; children begin a consistent pattern of improvements in BMIz during the school year and significant increases in BMIz during summer, with summertime increases in BMIz contributing to increased risk of becoming overweight or obese by 5th grade. Shortened sleep duration and circadian rhythm disturbances have been clearly implicated in obesity onset. Specifically, shifts in the daily behavioral rhythms of sleep and meal patterns are associated with increased adiposity, mediated through subsequent alterations in the molecular circadian clock. The transition from the school year to summer vacation represents a time during which children may experience changes in their behavioral rhythms (i.e., sleep and behavioral rhythms). Interpersonal Social Rhythm Therapy (IPSRT) is an evidence based treatment for bipolar disorder that has demonstrated efficacy to prevent the reoccurrences of mood disturbances by preventing the misalignment of endogenous circadian rhythms through the promotion of stable behavioral rhythms (e.g., sleep and meal patterns). The goals of the proposed research are to 1) adapt IPSRT to promote stable behavioral rhythms in children during summer vacation for the prevention of summer increases in BMIz (i.e., the rhythm project) and 2) conduct a pilot study to examine the feasibility, acceptability, and preliminary efficacy of the i♥rhythm project to prevent increases in children's BMIz during summer. mHealth technologies will be utilized in order to reach parents using a familiar and convenient resource during out of school times. The K99 phase will provide additional training in order to develop expertise in sleep and circadian rhythms research and the development of behavioral health interventions using mHealth technologies, as well as additional professional and research development needed to address gaps in training. The additional training will facilitate transition to a career focused on the development of behavioral interventions to address the role of sleep and circadian rhythms in the onset of childhood obesity. The proposed research addresses several gaps in scientific knowledge and practice: 1) there are a paucity of interventions addressing summer weight gain in children 2) there is a significant gap in knowledge regarding the role of sleep and circadian rhythms in the prevention of child obesity and 3) addressing summer weight gain provides an important opportunity to address the prevention of child obesity. This innovative application represents a novel application of an intervention with established efficacy to promote stable behavioral rhythms for treatment of bipolar disorder and promises to do the same for the prevention of child obesity. This research is the first to address the role of sleep and circadian rhythms in the prevention of child obesity and to examine the preliminary evidence of such an intervention on the long-term prevention of obesity.

PUBLIC HEALTH RELEVANCE: Summer is a time during which children's risk of obesity increases substantially, yet there is a paucity of interventions to prevent summer increases in BMI. Disrupted sleep patterns and behavioral rhythms, which may occur in the transition from the school year to summer vacation, have been implicated in the onset of obesity. The i♥rhythm project, a parent-child intervention targeting behavioral rhythms during summer for the prevention of obesity in young

elementary school children, will address a critical gap in knowledge regarding the role of sleep and behavioral rhythms in the prevention of obesity and fulfill an important need for interventions to address summer increases in BMI.

CRITIQUE NOTE: The sections that follow are the essentially unedited, verbatim comments of the reviewers assigned to this application. They are provided to illustrate the range of opinions expressed. The application was discussed and scored by all reviewers present. The attached commentaries may not necessarily reflect the position of the reviewers at the close of group discussion, nor the final majority opinion of the group. The Resume and Summary of Discussion, however, is the authoritative representation of the final outcome of the group discussion.

CRITIQUE 1:

Candidate: 2
Career Development Plan/Career Goals /Plan to Provide Mentoring: 2
Research Plan: 4
Mentor(s), Co-Mentor(s), Consultant(s), Collaborator(s): 1
Environment Commitment to the Candidate: 1

Overall Impact:

This a K99 career development award from a highly qualified candidate. The application is based on data from the PI and colleagues showing that increases in BMI z-scores occur during the summer months and therefore this time period is a target for weight reduction efforts. There are some strengths of the application including the novelty of the approach to target sleep and behavioral rhythms during summer for the prevention of obesity and the use of the Interpersonal and Social Rhythm Therapy (IPSRT) for preventing obesity. Other strengths include the mentoring team and the environment. Moderate weaknesses lie in the research plan.

1. Candidate:

Strengths

- Janette Moreno, PhD is an instructor in the Children’s Nutrition Research Center at Baylor College of Medicine. She is a clinical psychologist with training in pediatric obesity prevention and school-based interventions which will serve her well to lead these investigations.
- She completed an MA and PhD at LSU in clinical psychology with a minor in Public Health Care Administration.
- She has multiple publications related to this area of research
- Funding is through and industry grant to support multi-center RCT to compare impact of 2 dietary approaches to weight management on initial weight loss in adults; Foundation grant to examine factors related to summer weight gain in children. She is the coordinator or co-investigator on multiple previous grants.

Weaknesses

- The PI will devote 12 calendar months in years 1 and 2 to this project which leaves no time for any other career development or research efforts not related to this project.

2. Career Development Plan/Career Goals & Objectives/Plan to Provide Mentoring:

Strengths

- The candidate is seeking training on the role of sleep and behavioral rhythms on the development of childhood obesity. She is seeking to obtain training in 1. sleep research, 2. technology to deliver behavioral health interventions (mobile health technology), 3. advanced statistical methods and research design, professional career development.
- She will obtain training in actigraphy, polysomnography; spend 2-weeks at Rush University to be trained in measurement and analysis of sleep/wake patterns and salivary dim light melatonin onset; 5-day course in chronobiology and sleep medicine in Oxford; NIH week-long immersive program in use of mobile health technology; 1-week course at UAB on research designs and techniques for establishing causal inferences in behavioral obesity research; training in analysis of longitudinal data and advanced research design; attend national Scientific Meetings (3 per year).
- R00 phase will examine the feasibility, acceptability and preliminary efficacy of the intervention developed during K99 phase and secure a tenured track faculty position.
- Goal is to develop an R01 grant for a RCT examining efficacy of the behavioral intervention developed through the CDA for long-term prevention of child obesity.

Weaknesses

- The candidate proposes an ambitious travel schedule to attend week-long seminars, national conferences and other training.
- A schedule (table 6) is provided but doesn't provide plans/information for winter term.

3. Research Plan:

Strengths

- There is an increase in standardized BMIz during the summer months in children in the US
- The investigators performed a 5-year longitudinal study that examined BMI changes among 7600 children and found that on average children increased their BMIz during the summer and significantly decreased BMIz during school year.
- Circadian rhythm misalignment is implicated as a risk factor in obesity onset and summer is a time where sleep-wake cycles are altered. But little research is conducted during this time.
- Therefore, instilling consistent summertime family routines and parenting practices that support healthy sleep habits is important for child obesity prevention
- Interpersonal and social rhythm therapy (IPSRT) is an evidence-based treatment for bipolar disorder that promotes regularity of behavioral rhythms to prevent desynchronization of endogenous circadian rhythms that precede depressive and manic episodes. The content will adapt IPSRT components 1. Sleep hygiene, 2. Bedtime routines 3. Physical activity 4. Planning for and adapting to changes in routines and 5. Planning for summer vacation
- Plan to develop the i-heart-rhythm project, an adaptation of IPSRT, to promote healthy sleep habits and stable behavioral rhythms during summer for the prevention of obesity in young elementary school age children and examine the feasibility, acceptability and preliminary efficacy of the i-heart-rhythm project on the prevention of summer increases in BMIz.
- K99 specific aim 1: to adapt IPSRT to promote healthy sleep and stable behavioral rhythms in children for the prevention of increased BMIz during summer (yrs 1 and 2). To develop the mHealth delivery modes. The methods will use 3 delivery modes – 1. Electronic daily diary with automated feedback, 2. Social media content (Facebook) and 3. Book sharing intervention. The intervention will begin in the spring.

- Will adapt IPSRT for 5-8-year-old children to prevent summer increases in BMI by promoting parenting practices and family routines to maintain stable behavioral rhythms and encourage healthy sleep habits. Subjects will be stratified by gender, obesity status and socioeconomic status.
- Using Qualtrics is a strength. Qualtrics will be used to collect data from participants in real time and provide tailored automatic feedback
- Plan to recruit 30 5-8-year-old children in K-2rd grades and their parents
- R00 Specific aim 2: to conduct a pilot study to assess the feasibility, acceptability and preliminary efficacy of the i-heart-rhythm project to prevent increases in BMIz among 5-8-year-old children
- Plan to use a 2 group cluster randomized control design with randomization occurring after baseline and 5 evaluation periods (baseline, end of school year, end of intervention, and 9 and 12 mos. post intervention). This approach will explore the impact of the intervention on change in BMIz during the following summer.
- Will recruit up to 4 schools and recruit evenly across schools.
- The control group will only receive assessments. Plan for 36/group.
- The study is not powered to detect differences in outcome variables. A fully powered RCT will be proposed for the R01 application.

Weaknesses

- Meaningful data may be hard to obtain with the low sample number and the number of stratification variables used.
- It is not clear whether the summer increase in BMIz leads to worse long-term outcomes, especially if during the school year the BMI decreases.
- Preliminary studies are done with 300 parents who underwent semi-structured interviews on parenting practices and routines but how this data will be incorporated into the current application is not clear.
- One of the interventions is providing resources for families, questionnaires, etc. It is not clear how a list of resources for summer activities will help. There are many factors including cost/travel/time that may factor into whether a child participates in day camps, sports camps or other activities in the summer.
- The application lacks a discussion on alternative plans. What if the aims are not achieved?
- The impact of the intervention will be assessed during the following summer; ~ 1year after the intervention has started. Therefore, many variables come into play and could affect the outcome.
- Pilot study is not powered to detect differences in outcome variables

4. Mentor(s), Co-Mentor(s), Consultant(s), Collaborator(s):

Strengths

- There is an appropriate mentoring team led by Dr. Baranowski.
- Thomas Baranowski, PhD is the primary mentor. He is a Professor of Pediatrics in the Children's Nutrition Research Center at Baylor. He is an applied behavioral scientist with training in social psychology. He was the primary mentor for 14 post-docs and currently four fellows. He is Editor in Chief of Games for Health journal. He holds an R01 and USDA grants as

PI and co-I on R44. He has been the PI for many projects involving school children and their families regarding diet and physical activity.

- Deborah Thompson, PhD is a co-mentor. She is an Associate Professor of Pediatrics Nutrition at Baylor and a research nutritionist. Her research focuses on prevention of obesity and related diseases in youth through promotion of healthy diet and physical activity. She has expertise in qualitative methods and the use of behavioral theory in technology-based interventions. She has experience using digital media to reach youth to maintain change. She will provide mentorship on technology based intervention development and design, qualitative research methods and analysis, career development, ethics.
- Mentorship committee: Dr. Candice Alfano, PhD is a tenured Associate Professor in the Department of Psychology at the University of Houston and is the Director of the Sleep and Anxiety Center of Houston at the University of Houston. Her research interests include the role of early sleep processes in the etiology and development of emotion regulation and affective disorders in youth. Developed web-based behavioral sleep intervention for children. Will provide mentorship in the areas of pediatric sleep medicine and cognitive behavioral treatments for youth.
- Stephanie Crowley, PhD, is an Associate Professor in the Department of Behavioral Sciences at Rush University. She has expertise in studying sleep and circadian timing. She holds 2 large R01s.
- E. O'Brian Smith, PhD. is a biostatistician at Baylor and will provide research support.

Weaknesses

- none

5. Environment and Institutional Commitment to the Candidate:

Strengths

- The investigations will take place at the Baylor College of Medicine which houses The Children's Nutrition Research Center.
- Fort Bend Independent School District school system is the recruitment and school data collection site. It was identified as the most culturally diverse county in US.
- Letter of support by Dr. Mark Kline, Chairman Department of Pediatrics

Weaknesses

- none

Protections for Human Subjects:

- Acceptable Risks and Adequate Protections

Data and Safety Monitoring Plan (Applicable for Clinical Trials Only):

- Acceptable

Inclusion of Women, Minorities and Children:

- Sex/Gender: Distribution justified scientifically
- Race/Ethnicity: Distribution justified scientifically
- For NIH-Defined Phase III trials, Plans for valid design and analysis: Not applicable
- Inclusion/Exclusion of Children under 18: Including ages < 18; justified scientifically

Vertebrate Animals:

- Not Applicable (No Vertebrate Animals)

Biohazards:

- Not Applicable (No Biohazards)

Training in the Responsible Conduct of Research:

- Acceptable

Comments on Format (Required):

- acceptable

Comments on Subject Matter (Required):

- acceptable

Comments on Faculty Participation (Required; not applicable for mid- and senior-career awards):

- acceptable

Comments on Duration (Required):

- acceptable

Comments on Frequency (Required):

- acceptable

Resource Sharing Plans:

- Acceptable

Budget and Period of Support:

- Recommend as Requested

CRITIQUE 2:

Candidate: 1
Career Development Plan/Career Goals /Plan to Provide Mentoring: 1
Research Plan: 4
Mentor(s), Co-Mentor(s), Consultant(s), Collaborator(s): 2
Environment Commitment to the Candidate: 1

Overall Impact:

This grant is submitted by an outstanding candidate and proposes work related to sleep disturbance and excess weight gain in children during the summer time period. The grant has many strong components as listed below; however enthusiasm was diminished by the extensive focus on complicated method adaption in the face of unclear certainty if changing sleep behavior and circadian rhythms is causal in the summer weight gain. The project is lacking consideration for controlling for

other behaviors and environmental influences that likely combine to create increased summer weight gain.

1. Candidate:

Strengths

- Instructor of Pediatrics at Baylor College of Medicine
- Impressive track record of clinical research including large RCTs
- Excellent publication record

Weaknesses

- none

2. Career Development Plan/Career Goals & Objectives/Plan to Provide Mentoring:

Strengths

- Outstanding development plan with didactics and workshops and conferences plus research design to position her as an expert in sleep as it relates to childhood obesity
- Excellent mentoring plan with appropriate complimentary expertise
- Well-conceived transition to independent portion of the grant

Weaknesses

- none

3. Research Plan:

Strengths

- Focus on summer time as risk period for adiposity gain is novel and important area
- Although sleep is recognized as a contributor, few interventions exist to successfully improve sleep patterns in children

Weaknesses

- The hypotheses proposed are uninspiring. If it is a feasibility study, then it may not need a control group. If it is powered to show an effect, then there is benefit to having a control group.
- The work described in Aim 1 presupposes that there is a benefit to adapting the IPSRT to obesity prevention in children because sleep is causal in the excess weight gain seen in the summer in children. Are there other behaviors that differ in the summer compared to school time such as food choices and availability, screen time, and other sedentary time? How will these be controlled for?
- The adaption of IPSRT is extensive and complicated with multiple delivery methods planned. The complexity of the intervention will complicate the testing of the question – if improvement in sleep improves BMI.
- Requirement of written English skills to participate will exclude some important populations

4. Mentor(s), Co-Mentor(s), Consultant(s), Collaborator(s):

Strengths

- Outstanding mentor with long track record in related field
- Great mentor committee

Weaknesses

- none

5. Environment and Institutional Commitment to the Candidate:

Strengths

- Strong letters of support
- Commitment to transitioning Dr. Moreno to a tenure track position

Weaknesses

- none

Protections for Human Subjects:

- Acceptable Risks and Adequate Protections

Data and Safety Monitoring Plan (Applicable for Clinical Trials Only):

Inclusion of Women, Minorities and Children:

- Sex/Gender: Distribution justified scientifically
- Race/Ethnicity: Distribution justified scientifically
- For NIH-Defined Phase III trials, Plans for valid design and analysis:
- Inclusion/Exclusion of Children under 18:

Vertebrate Animals:

Budget and Period of Support:

- Recommended budget modifications or possible overlap identified:

CRITIQUE 3:

Candidate: 1
Career Development Plan/Career Goals /Plan to Provide Mentoring: 2
Research Plan: 2
Mentor(s), Co-Mentor(s), Consultant(s), Collaborator(s): 1
Environment Commitment to the Candidate: 1

Overall Impact:

This is a very well formulated and written proposal from an enthusiastic and productive investigator working at Baylor College of Medicine. With training in clinical psychology, she has served for many years as research and project coordinator, now she needs more training to make her transition to independent investigator possible. The research objective of this proposal is to evaluate interventions to prevent summer increases in BMI in children. The research plan is well designed, and will provide

valuable pilot data for the next phase of her career. The team of mentors is impressive, and she already published with some of them. The career plan is adequate for this stage of her career. She is expected to be successful in her career path and contribute valuable research to the field of obesity prevention in children.

1. Candidate:

Strengths

- Excellent publication records (29 publications listed)
- Recipient of a beginning Grant in Aid award from the American Heart
- Currently the PI of an award from the Mullen Foundation
- Extended exposure to various aspects of research (subjects recruitment, data handling, grant writing)
- Her career goals are clearly stated. The present grant logically follows the objectives she has established for her future development, both in terms of science and logistic.

2. Career Development Plan/Career Goals & Objectives/Plan to Provide Mentoring:

Strengths

- Training goals (sleep and behavioral rhythms measurement and research, use of technology to deliver behavioral health interventions, advanced statistical methods and research design, professional career development) are clearly defined and adequate.
- Good balance of training and research.

Weaknesses

- Maybe longer formal works (rather than short week long seminars) would be advisable

3. Research Plan:

Strengths

- Focusing on interventions during a time period of bigger risk for increasing BMIs (summer time) is significant to prevent obesity in children
- Innovative intervention to be tested
- Comprehensive battery of measurement will be performed to assess the circadian rhythm
- The plan is designed to provide feasibility data (for Interpersonal and Social Rhythm Therapy (IPSRT)) for a larger R01. This is valuable strategy.

4. Mentor(s), Co-Mentor(s), Consultant(s), Collaborator(s):

Strengths

- Mentor: Dr. Baranowski. Impressive list of 402 publications, adequate funding. Brings in expertise in behavioral health sciences
- Co-Mentor: Dr. Thompson. Excellent track records of publications. Brings in expertise in technology to deliver behavioral health interventions
- Collaborator: Dr. Alfano. Excellent publication, mentoring and funding records. Brings in expertise in assessing sleep

- Collaborator: Dr. Crowley. She provides hands-on experience with studying sleep and circadian timing in humans.
- Collaborator: Dr. Smith. He provides invaluable expertise in statistics and experiment design. Long experience in mentoring students, and impressive list of publications

5. Environment and Institutional Commitment to the Candidate:

Strengths

- One of six federally funded human nutrition research centers in the United States, the Children's Nutrition Research Center (CNRC) has strong commitment to research and strong capabilities to recruit (12000 subjects recruited since 1978)
- It was valuable to have a detailed description of her job duties. It was indeed surprising to see she is not currently post-doc.

Protections for Human Subjects:

- Acceptable Risks and Adequate Protections
- very careful plan, which reflects the long experience of the candidate with this type of research

Data and Safety Monitoring Plan (Applicable for Clinical Trials Only):

Not Applicable (No Clinical Trials)

Inclusion of Women, Minorities and Children:

- Sex/Gender: Distribution justified scientifically
- Race/Ethnicity: Distribution justified scientifically
- For NIH-Defined Phase III trials, Plans for valid design and analysis: Not applicable
- Inclusion/Exclusion of Children under 18: Including ages < 18; justified scientifically

Vertebrate Animals:

- Not Applicable (No Vertebrate Animals)

Biohazards:

- Not Applicable (No Biohazards)

Training in the Responsible Conduct of Research:

- Acceptable

Comments on Format (Required):

- formal training and in person training with the mentors

Comments on Subject Matter (Required):

- appropriate topics covered

Comments on Faculty Participation (Required; not applicable for mid- and senior-career awards):

- The responsible and ethical conduct of research is an interest of Dr. Thompson's

Comments on Duration (Required):

- Fundamentals of Clinical Investigation (Year 1), 12 hours
- Research Compliance Courses and Workshops (Year 1 and 2) are 5.5Hrs
- Collaborative Institutional Training Initiative (CITI) Online Training in Human Subjects Research (Year 2).

Comments on Frequency (Required):

- Fundamentals of Clinical Investigation (Year 1), 3 hour session weekly for 1 month
- Research Compliance Courses and Workshops (Year 1 and 2) 1 hour monthly
- Collaborative Institutional Training Initiative (CITI) Online Training in Human Subjects Research (Year 2) - renewed as needed

Resource Sharing Plans:

- Acceptable

Budget and Period of Support:

- Recommend as Requested

Additional Comments to Applicant (Optional):

- Some inaccuracies in the list of publications of the candidate. Also, is really only one publication funded by NIH (i.e., no PMCID)?
- No role stated in the personal statement of the biosketch of Dr. Baranowski.
- Few grant should be listed as completed in the bio of Dr. Thompson
- No complete list of publications is provided by Dr. Alfano and for Dr. Smith, contrary to instructions. Also, all biosketches do not conform to the updated standard: they should include the expiration date of the template on the top right of the header.

THE FOLLOWING SECTIONS WERE PREPARED BY THE SCIENTIFIC REVIEW OFFICER TO SUMMARIZE THE OUTCOME OF DISCUSSIONS OF THE REVIEW COMMITTEE, OR REVIEWERS' WRITTEN CRITIQUES, ON THE FOLLOWING ISSUES:

PROTECTION OF HUMAN SUBJECTS (Resume): ACCEPTABLE

INCLUSION OF WOMEN PLAN (Resume): ACCEPTABLE

INCLUSION OF MINORITIES PLAN (Resume): ACCEPTABLE

INCLUSION OF CHILDREN PLAN (Resume): ACCEPTABLE

COMMITTEE BUDGET RECOMMENDATIONS: The budget was recommended as requested.

TRAINING IN THE RESPONSIBLE CONDUCT OF RESEARCH: ACCEPTABLE.

Footnotes for 1 K99 HD091396-01; PI Name: Moreno, Jennette P

NIH has modified its policy regarding the receipt of resubmissions (amended applications). See Guide Notice NOT-OD-14-074 at http://grants.nih.gov/grants/guide/notice-files/NOT-OD-14-074.html. The impact/priority score is calculated after discussion of an application by averaging the overall scores (1-9) given by all voting reviewers on the committee and multiplying by 10. The criterion scores are submitted prior to the meeting by the individual reviewers assigned to an application, and are not discussed specifically at the review meeting or calculated into the overall impact score. Some applications also receive a percentile ranking. For details on the review process, see http://grants.nih.gov/grants/peer_review_process.htm#scoring.

MEETING ROSTER
Pediatrics Subcommittee
National Institute of Child Health and Human Development Initial Review Group
EUNICE KENNEDY SHRIVER NATIONAL INSTITUTE OF CHILD HEALTH & HUMAN DEVELOPMENT

CHHD-A
10/20/2016 - 10/21/2016

CHAIRPERSON(S)

NOTTERMAN, DANIEL A., MD
SENIOR RESEARCH SCHOLAR
LEWIS THOMAS LABORATORY
DEPARTMENT OF MOLECULAR BIOLOGY
PRINCETON UNIVERSITY
PRINCETON, NJ 08544

MEMBERS

ANEJA, RAJESH K, MBBS, MD *
ASSOCIATE PROFESSOR
CRITICAL CARE MEDICINE
UNIVERSITY OF PITTSBURGH SCHOOL OF MEDICINE
PITTSBURGH, PA 15224

CAMPBELL, DEBORAH, MD
PROFESSOR
DEPARTMENT OF NEONATOLOGY
CHILDREN'S HOSPITAL AT MONTEFIORE
ALBERT EINSTEIN COLLEGE OF MEDICINE
BRONX, NY 10461

CORNFIELD, DAVID N., MD
PROFESSOR IN PEDIATRIC PULMONARY MEDICINE
DIRECTOR, CENTER FOR EXCELLENCE
IN PULMONARY BIOLOGY
STANFORD UNIVERSITY SCHOOL OF MEDICINE
STANFORD, CA 94304

DENNERY, PHYLLIS A., MD
PROFESSOR AND SYLVIA K. HASSENFELD CHAIR OF PEDIATRICS
PROFESSOR OF MOLECULAR BIOLOGY CELL BIOLOGY & BIOCHEMISTRY
BROWN UNIVERSITY
PEDIATRICIAN IN CHIEF HASBRO CHILDREN'S HOSPITAL
PROVIDENCE, RI 02903

FLANAGAN, JOHN M JR, PHD *
PROFESSOR
DEPARTMENT OF BIOCHEMISTRY
AND MOLECULAR BIOLOGY
PENN STATE COLLEGE OF MEDICINE
HERSHEY, PA 17036

GANGWANI, LAXMAN DASS, PHD *
ASSOCIATE PROFESSOR
BIOMEDICAL SCIENCES
PAUL L. FOSTER SCHOOL OF MEDICINE
TEXAS TECH UNIVERSITY HEALTH SCIENCES CENTER
EL PASO
EL PASO , TX 79912

GREEN, THOMAS P., MD *
PROFESSOR AND CHAIR
DEPARTMENT OF PEDIATRICS
NORTHWESTERN UNIVERSITY
CHILDREN'S MEMORIAL HOSPITAL
CHICAGO, IL 60614

HAGADORN, JAMES I., MD *
ASSISTANT PROFESSOR OF PEDIATRICS
DIVISION OF NEONATOLOGY
CONNECTICUT CHILDREN'S MEDICAL CENTER
HARTFORD, CT 06106

HUNT, CARL ELMER, MD *
PROFESSOR
DEPARTMENT OF PEDIATRICS
UNIFORMED SERVICES UNIVERSITY OF HEALTH SCIENCES
BETHESDA, MD 20892

KAPLAN, JENNIFER MELISSA, BA, MD, MS
ASSOCIATE PROFESSOR
DIVISION OF CRITICAL CARE MEDICINE
CINCINNATI CHILDREN'S HOPITAL MEDICAL CENTER
CINCINNATI, OH 45229

KOENIG, MARY KAY, MD
ASSOCIATE PROFESSOR
DIVISION OF CHILD & ADOLESCENT NEUROLOGY
DEPARTMENT OF PEDIATRICS
UNIVERSITY OF TEXAS, HOUSTON
HOUSTON, TX 77030

LIECHTY, EDWARD A., MD
PROFESSOR
DEPARTMENT OF PEDIATRICS
JAMES W. RILEY HOSPITAL FOR CHILDREN
INDIANA UNIVERSITY SCHOOL OF MEDICINE
INDIANAPOLIS, IN 46202

MANGIA, SILVIA, PHD *
ASSOCIATE PROFESSOR
DEPARTMENT OF RADIOLOGY
CENTER FOR MAGNETIC RESONANCE RESEARCH
UNIVERSITY OF MINNESOTA
MINNEAPOLIS, MN 55455

NIGROVIC, LISE E., MD, MPH
ASSOCIATE PROFESSOR OF PEDIATRICS
DIVISION OF EMERGENCY MEDICINE
BOSTON CHILDREN'S HOSPITAL
HARVARD MEDICAL SCHOOL
BOSTON, MA 02115

PAUL, IAN A., PHD
PROFESSOR
DEPARTMENT OF PSYCHIATRY AND HUMAN BEHAVIOR
AND NEUROBIOLOGY AND ANATOMICAL SCIENCES
UNIVERSITY OF MISSISSIPPI MEDICAL CENTER
JACKSON, MS 39216

POLLACK, MURRAY MICHAEL, MBA, MD
PROFESSOR OF PEDIATRICS
PROFESSOR OF PEDIATRICS
CHILDREN'S NATIONAL HEALTH SYSTEM
GEORGE WASHINGTON UNIVERSITY SCHOOL OF MEDICINE
AND HEALTH SCIENCES
WASHINGTON, DC 20010

SAPIENZA, CARMEN, PHD
PROFESSOR
FELS INSTITUTE FOR CANCER RESEARCH
PROFESSOR, DEPARTMENT OF PATHOLOGY
AND LABORATORY MEDICINE
TEMPLE UNIVERSITY SCHOOL OF MEDICINE
PHILADELPHIA, PA 19140

SHAFFER, MICHELE L, PHD *
ASSOCIATE PROFESSOR
SEATTLE CHILDREN'S RESEARCH INSTITUTE
UNIVERSITY OF WASHINGTON
SEATTLE, WA 98101

SOLER ALFONSO, CLAUDIA
GENETICS CENTER
COOK CHILDREN'S PHYSICIAN NETWORK
FORT WORTH, TX 76102

SWAMY, GEETA K., MD
ASSOCIATE PROFESSOR
DEPARTMENT OF OBSTETRICS AND GYNECOLOGY
DUKE UNIVERSITY SCHOOL OF MEDICINE
DURHAM, NC 27705

TIAO, GREGORY M, MD *
PROFESSOR OF PEDIATRIC AND THORACIC SURGERY
CINCINNATI CHILDRENS HOSPITAL AND MEDICAL CENTER
CINCINNATI, OH 45229

VOS, MIRIAM B., MD
ASSOCIATE PROFESSOR OF PEDIATRICS
DIVISION OF GASTROENTEROLOGY,
HEPATOLOGY, AND NUTRITION
EMORY UNIVERSITY SCHOOL OF MEDICINE
ATLANTA, GA 30322

WANG, KASPER SAONUN, MD
ASSOCIATE PROFESSOR OF SURGERY
KECK SCHOOL OF MEDICINE
UNIVERSITY OF SOUTHERN CALIFORNIA
DIVISION OF PEDIATRIC SURGERY
CHILDREN'S HOSPITAL LOS ANGELES
LOS ANGELES, CA 90027

WEINSTEIN, DAVID A, MD
PROFESSOR AND DIRECTOR
GLYCOGEN STORAGE DISEASE PROGRAM
UNIVERSITY OF FLORIDA COLLEGE OF MEDICINE
GAINSVILLE, FL 32610

SCIENTIFIC REVIEW OFFICER

ANAND, RITA, PHD
SCIENTIFIC REVIEW OFFICER
DIVISION OF SCIENTIFIC REVIEW
NATIONAL INSTITUTE OF CHILD HEALTH
AND HUMAN DEVELOPMENT, NIH
6100 EXECUTIVE BLVD. ROOM 5B01
BETHESDA, MD 20892

EXTRAMURAL SUPPORT ASSISTANT

SILVERMAN, STEVEN D.
EXTRAMURAL SUPPORT ASSISTANT
SCIENTIFC REVIEW BRANCH
EUNICE KENNEDY SHRIVER NATIONAL INSTITUTE OF
CHILD HEALTH AND HUMAN DEVELOPMENT
6710 B ROCKLEDGE DRIVE, ROOM 2131A
BETHESDA, MD 20892-7510

* Temporary Member. For grant applications, temporary members may participate in the entire meeting or may review only selected applications as needed.

Consultants are required to absent themselves from the room during the review of any application if their presence would constitute or appear to constitute a conflict of interest.
